# Dynamic Changes of Functional Pain Connectome in Women with Primary Dysmenorrhea

**DOI:** 10.1038/srep24543

**Published:** 2016-04-19

**Authors:** Ting-Hsuan Wu, Cheng-Hao Tu, Hsiang-Tai Chao, Wei-Chi Li, Intan Low, Chih-Ying Chuang, Tzu-Chen Yeh, Chou-Ming Cheng, Chih-Che Chou, Li-Fen Chen, Jen-Chuen Hsieh

**Affiliations:** 1 Institute of Brain Science, School of Medicine, National Yang-Ming University, Taipei, Taiwan; 2 Integrated Brain Research Unit, Division of Clinical Research, Department of Medical Research, Taipei Veterans General Hospital, Taipei, Taiwan; 3 Department of Obstetrics and Gynecology, School of Medicine, National Yang-Ming University, Taipei, Taiwan; 4 Department of Obstetrics and Gynecology, Taipei Veterans General Hospital, Taipei, Taiwan; 5 Institute of Biomedical Informatics, School of Medicine, National Yang-Ming University, Taipei, Taiwan

## Abstract

Primary dysmenorrhea (PDM) is the most prevalent gynecological problem. Many key brain systems are engaged in pain processing. In light of dynamic communication within and between systems (or networks) in shaping pain experience and behavior, the intra-regional functional connectivity (FC) in the hub regions of the systems may be altered and the functional interactions in terms of inter-regional FCs among the networks may be reorganized to cope with the repeated stress of menstrual pain in PDM. Forty-six otherwise healthy PDM subjects and 49 age-matched, healthy female control subjects were enrolled. Intra- and inter-regional FC were assessed using regional homogeneity (ReHo) and ReHo-seeded FC analyses, respectively. PDM women exhibited a *trait-related* ReHo reduction in the ventromedial prefrontal cortex, part of the default mode network (DMN), during the periovulatory phase. The *trait-related* hypoconnectivity of DMN-salience network and hyperconnectivity of DMN-executive control network across the menstrual cycle featured a dynamic transition from affective processing of pain salience to cognitive modulation. The altered DMN-sensorimotor network may be an ongoing representation of cumulative menstrual pain. The findings indicate that women with long-term PDM may develop adaptive neuroplasticity and functional reorganization with a network shift from affective processing of salience to the cognitive modulation of pain.

Primary dysmenorrhea (PDM), or menstrual pain without organic causes, is the most prevalent menstrual complaint of young females. Between 40 to 90% of female adolescents have experienced PDM and 10–20% describe their suffering as so severe and distressing that it requires absence from school or work^1^. Notably, otherwise healthy women with PDM often display a higher prevalence of incidental brain findings^2^, particularly of normal variants, when compared with control subjects. Additionally, genetic factors, such as the *brain-derived neurotrophic factor (BDNF)* Val66Met polymorphism, may account for the susceptibility of an individual to PDM^3^.

We have previously reported that long-term PDM is associated with alterations in brain metabolism and functional connectivity (FC) in pain modulatory systems, as well as in *state*- and *trait-related* structures^4^,^5^,^6^,^7^. *State-related* changes are menstrual pain-primed, whereas *trait-related* changes exist even without symptoms. Notably, pain-related regions, such as the medial prefrontal cortex (mPFC), posterior cingulate cortex and insula, exhibit abnormal functional and structural changes in otherwise healthy women with PDM^4^,^6^,^8^. These regions are parts of the pain matrix^9^ and are associated with cognitive control of emotions, self-referential processing^10^, contextualizing^11^,^12^ and pain generation and modulation^13^,^14^.

Many key brain systems are engaged for menstrual pain processing; many key regions of these systems are also neural substrates of pain matrix that participate substantially in central processing of pain. Recent studies have demonstrated that the FC of large-scale brain systems/networks may be dynamically altered in patients with PDM and other chronic pain disorders and that these alterations can be either adaptive or maladaptive^7^,^15^,^16^. Such altered cross-network connectivity and the imbalance between the systems have been suggested to be a principal pain-related brain abnormality in chronic pain patients^17^. The default mode network (DMN) plays an important role in pain disorders^15^,^18^. It not only participates in episodic memory and in intrinsic attention but also modulates the experience of pain^18^,^19^. The DMN and sensorimotor network (SMN) reorganize in patients with arm amputation that can be with and without phantom pain^20^. The mPFC, a central node within the DMN, acts as a cardinal coordinator by interacting with the dorsal attention network (DAN), executive control network (ECN) and salience network (SN) during stress and pain adaptation and modulation^15^,^21^,^22^. The ECN balances self-referential awareness with attention to salient information^23^ and modulates pain anticipation^24^. The SN, which is related to anxiety^25^, is activated when attention is maintained on pain^15^. The DAN provides a direct corticocortical pathway for the attentional modulation of pain^22^. The DMN, ECN and SN networks constitute the core neural systems in the human brain^26^. To address the resilient crosstalk between these systems under clinical pain condition, PDM as a genuine condition of chronic pain^7^ with spontaneous alternation of painful and pain-free periods may serve as a good clinical model of choice. As pain experiences in females are usually more sophisticated than males^27^ and PDM subjects are vulnerable to anxiety^28^, it is plausible the FC alterations in PDMs may be manifested as *trait-related* changes in response to different affective states. Furthermore, PDM may feature functional interactions between large-scale brain networks.

In this study, we implemented a hierarchical approach to study the FC constructs of PDM. We reasoned that FC changes could be manifested as intra-regional (local) and inter-regional (long- distance) FC changes. To study the neuroplasticity of intra-regional FC, we used regional homogeneity (ReHo) analyses to estimate the local synchronization of resting brain activity within a segregated brain region^29^. An increased local synchrony indicates increased coherence of spontaneous neuronal activity^30^, which implicates functional integration within a region. Conversely, desynchronized local activity indicates divergent local functions or increased intra-regional functional segregation^31^. To study inter-regional FC alterations among the aforementioned networks, we performed seed-based FC analyses of large-scale networks in the significant regions that were identified in the ReHo analysis. Incorporating ReHo in the seed-based FC analyses may improve the sensitivity of FC analyses and reduce the uncertainty of seed selection^32^. We considered the regions of ReHo alterations the key functional hubs for PDM, and the changes of inter-regional FCs as seeded on these ReHo-hubs may serve as critical functional correlates for brain resilience under long-term PDM. Collectively, we hypothesized that the local synchronization within hub region(s) of the key systems, particularly the DMN, would be altered and that the functional interactions among the networks (DMN, SN, ECN, and DAN) would be dynamically and functionally reorganized in patients with PDM to cope with the repeated stress of menstrual pain.

## Results

### Demographic and psychological data

In brief, 46 PDMs and 49 controls (CON) were enrolled in the current neuroimaging study. There were no significant between-group differences regarding age (PDM: 23.33 ± 2.42 years of age, CON: 23.80 ± 2.47 years of age, *p* = 0.351), age at menarche (PDM: 11.93 ± 1.32 years of age, CON: 12.31 ± 1.08 years of age, *p* = 0.14), years of menstruating (PDM: 11.39 ± 2.84 years, CON: 11.49 ± 2.86, *p* = 0.867), or average duration of one menstrual cycle (PDM: 29.50 ± 1.28 days, CON: 29.52 ± 1.19 days, *p* = 0.936). For the menstrual pain experience, PDM subjects had in average 9.22 ± 2.85 years of pain history; 65.2% of them had absenteeism from school or works and 41.3% of them had taken analgesics occasionally. The PDM group had been experiencing moderate cyclic menstrual pain (present total scores of McGill Pain Questionnaire-pain rating index: 30.11 ± 11.99, with sensory score: 16.54 ± 6.01, affective score: 3.91 ± 2.85, evaluation score: 2.54 ± 2.02, miscellaneous score: 7.11 ± 3.62 in each category; present pain intensity: 2.72 ± 0.96).

For the psychological inventories, significant group (PDM vs. CON)-phase (menstruation [MENS] vs. periovulatory [POV]) interactions were found in the total (*F* = 5.921, *R*^*2*^ = 0.060, *p* = 0.017) and helplessness scores (*F* = 8.498, *R*^*2*^ = 0.084, *p* = 0.004) of the Pain Catastrophizing Scale (PCS), Beck Anxiety Inventory (BAI; *F* = 43.802, *R*^*2*^ = 0.320, *p* < 0.001), the state anxiety (*F* = 20.055, *R*^*2*^ = 0.177, *p* < 0.001) of Spielberger State-Trait Anxiety Inventory (STAI), and Beck Depression Inventory (BDI; *F* = 31.910, *R*^*2*^ = 0.255, *p* < 0.001). No significant group-phase interactions were found in the rumination (*F* = 1.569, *R*^*2*^ = 0.017, *p* = 0.214) and magnification (*F* = 2.021, *R*^*2*^ = 0.021, *p* = 0.158) scores of the PCS, and trait anxiety of STAI (*F* = 2.892, *R*^*2*^ = 0.030, *p* = 0.092). Significant main effects of group were found in the total (*F* = 71.504, *R*^*2*^ = 0.435, *p* < 0.001), rumination (*F* = 75.315, *R*^*2*^ = 0.447, *p* < 0.001), helplessness (*F* = 68.698, *R*^*2*^ = 0.425, *p* < 0.001) and magnification (*F* = 31.026, *R*^*2*^ = 0.250, *p* < 0.001) scores of the PCS, BAI (*F* = 57.994, *R*^*2*^ = 0.384, *p* < 0.001), the state anxiety (*F* = 16.437, *R*^*2*^ = 0.150, *p* < 0.001) and trait anxiety (*F* = 8.312, *R*^*2*^ = 0.083, *p* = 0.005) of STAI, and BDI (*F* = 14.514, *R*^*2*^ = 0.135, *p* < 0.001). Significant main effects of phase were found in the BAI (*F* = 27.334, *R*^*2*^ = 0.227, *p* < 0.001), state anxiety (*F* = 23.071, *R*^*2*^ = 0.199, *p* < 0.001) of STAI and BDI (*F* = 20.724, *R*^*2*^ = 0.182, *p* < 0.001). No main effects of phase were found in the total (*F* = 3.276, *R*^*2*^ = 0.034, *p* = 0.074), rumination (*F* = 2.544, *R*^*2*^ = 0.027, *p* = 0.114), helplessness (*F* = 3.713, *R*^*2*^ = 0.038, *p* = 0.057) and magnification (*F* = 0.264, *R*^*2*^ = 0.003, *p* = 0.609) scores of the PCS, and the trait anxiety (*F* = 1.518, *R*^*2*^ = 0.016, *p* = 0.221) of STAI (see Supplementary Table S1).

### Serum hormone levels

There were no significant between-group differences regarding the levels of estradiol, progesterone or testosterone in MENS phase and POV phase, respectively (see Supplementary Table S2).

### Intra-regional connectivity (ReHo)

**Figure 1:**
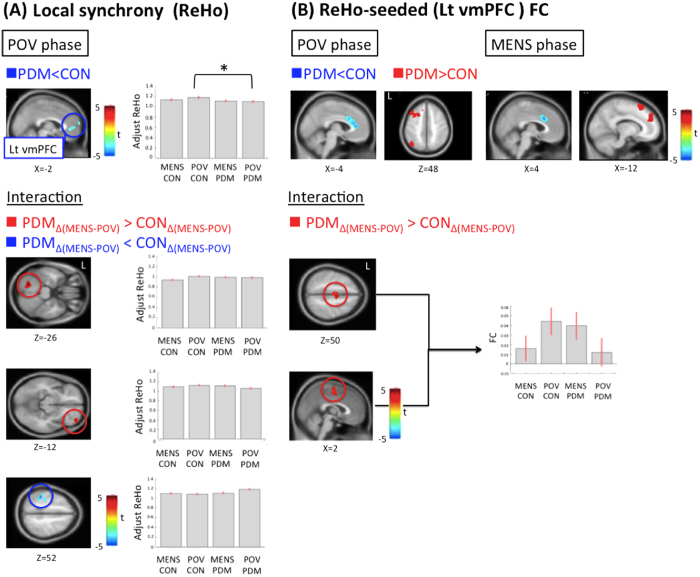
Statistical maps of ReHo, ReHo-seeded (Lt vmPFC) FC and the interaction effects. (**A**) *ReHo maps.* PDM group shows decreased ReHo of the Lt vmPFC during the POV phase when comparing to the CON group. For the interaction effect, the between-group (PDM vs. CON) comparison of phase differences (MENS vs. POV) reveals significant greater ReHo alterations in the Lt declive and right orbitofrontal cortex (OFC), and lesser ReHo alterations in the Lt anterior intraparietal sulcus and superior parietal lobule. The bar charts show adjusted ReHo values at the peak voxel of the vmPFC. The error bar corresponds to a 90% confidence interval. (**B**) *FC maps.* For ReHo-seeded (Lt vmPFC) FC between-group comparisons, hypoconnectivity in the Lt vmPFC-dorsal anterior cingulate cortex FC and hyperconnectivity in the Lt vmPFC-dorsomedial prefrontal cortex FC are noted in the PDM group when compared to the CON group during both phases. Hypoconnectivity in the Lt vmPFC-pregenual anterior cingulate cortex FC and hyperconnectivity in the Lt vmPFC-dorsolateral prefrontal cortex and -ventral parietal lobe FCs are observed in the PDM group during the POV phase. The PDM group (vs. CON group) shows greater between-phase (MENS vs. POV) Lt vmPFC-supplementary motor area (SMA) FC. Red regions are associated with increased FC and greater phase-difference of ReHo or FC in the PDM group. Blue regions are associated with decreased ReHo, decreased FC and lesser phase-difference of ReHo or FC in the PDM group. Significance thresholded at the uncorrected voxel level p = 0.005 followed by the FWE-corrected cluster level p = 0.05. ReHo, regional homogeneity; PDM, primary dysmenorrhea; CON, control; MENS, menstrual phase; POV, periovulatory phase; FC, functional connectivity; vmPFC, ventromedial prefrontal cortex; Lt, left. *Denotes the contrast(s) that are significant in between-group comparisons.

**Figure 2:**
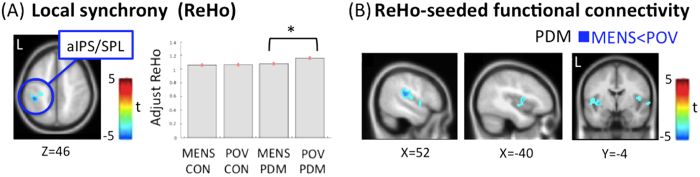
The *state-related* ReHo and FC alterations in PDM. (**A**) *ReHo maps*. PDM group shows decreased ReHo of the anterior part of the intraparietal sulcus (aIPS) and superior parietal lobe (SPL) during the MENS phase when compared to the POV phase. The bar charts indicate the adjusted ReHo values at the peak voxel of the aIPS/SPL. The error bar corresponds to a 90% confidence interval. (**B**) *FC maps.* For ReHo-seeded (aIPS/SPL) FC between-phase comparisons, hypoconnectivity in the aIPS-bilateral posterior insula and -right postcentral gyrus FCs are observed during the MENS phase in the PDM group. Blue regions are associated with either decreased ReHo or decreased FC. Significance thresholded at the uncorrected voxel level p = 0.005 followed by the FWE-corrected cluster level p = 0.05. *Denotes the contrast(s) that are significant in between-phase comparisons. For abbreviations, see the legend of Fig. 1.

For the interaction effect, the between-group comparison of phase differences revealed significant greater ReHo alterations in the left declive and right orbitofrontal cortex (OFC), and lesser ReHo alterations in the left aIPS/SPL in the PDM group (Table 1; Fig. 1A). No main effects of group and phase were noted. For the between-group planned contrast, there was no difference during the MENS phase. The bilateral ventromedial prefrontal cortex (vmPFC, BA10) of DMN network, exhibited a trait-related reduction of ReHo during the POV phase in the PDM group vs. CON group (Table 1; Figs 1A and S1). For the between-phase planned contrast, PDM group demonstrated *state-related* reduction of the ReHo in the anterior part of the left intraparietal sulcus (aIPS, BA40) and left superior parietal lobe (SPL) of DAN network during the MENS phase vs. the POV phase (Table 1; Figs 2A and S1). The CON group demonstrated *state-related* reduction of ReHo in the left declive during the MENS phase vs. the POV phase.

### Inter-regional connectivity (FC)

#### vmPFC (DMN network)

For the interaction effect, the PDM group as compared to the CON group exhibited greater left vmPFC (x = −6, y = 50, z = 4)-supplementary motor area (SMA) FC during MENS phase relative to POV phase (Table 2; Fig. 1B). No interaction was found when the right vmPFC (x = 6, y = 62, z = 22) was used as the seed. For the main effects, please refer to the Supplementary Table S3. For the between-group planned contrast, the PDM group as compared to the CON group exhibited hypoconnectivity of the left vmPFC-bilateral dorsal anterior cingulate cortex (dACC; substrate of SN network) circuit and hyperconnectivity of the left vmPFC-left dorsomedial prefrontal cortex (dmPFC; substrate of ECN network) circuit during both phases. In addition, the PDM group as compared to the CON group exhibited hypoconnectivity of the left vmPFC-bilateral pregenual anterior cingulate cortex (pACC; substrate of SN network) circuit and hyperconnectivity of the left vmPFC-left dorsolateral prefrontal cortex (DLPFC; substrate of ECN network) and left ventral parietal lobe (VPL; substrate of ECN network) circuits during the POV phase (Fig. 1B; Table 2; Figs S2, S3). Furthermore, both the right vmPFC-right inferior frontal gyrus (IFG; substrate of SN network) circuit and the right vmPFC-pACC circuit (DMN-DMN network) exhibited hypoconnectivity in the PDM group as compared to CON group during the POV phase (Table 2; Fig. S4). No between-group difference was detected during the MENS phase when the right vmPFC was used as the seed. For the between-phase planned contrast, no difference was found in the PDM group when the left vmPFC was seeded. In the CON group, hypoconnectivity of the left vmPFC-SMA circuit was found during the MENS phase as compared to the POV phase. In the PDM group, hypoconnectivity of the right vmPFC-pons circuit was found during the MENS phase as compared to the POV phase (Table 2). There was no between-phase difference in the CON group when the right vmPFC was seeded.

#### aIPS/SPL (DAN network)

For the interaction effect, no group-phase interaction was found when the left aIPS/SPL (x = −42, y = −36, z = 48) was seeded. For the main effects, please refer to the Supplementary Table S4. For the between-group planned contrast, the PDM group as compared to the CON group exhibited hyperconnectivity of the aIPS/SPL-MTG circuit and hypoconnectivity of the aIPS/SPL-anterior lobe of the cerebellum circuit during the MENS phase. The PDM group as compared to the CON group exhibited hyperconnectivity of the aIPS/SPL-MTG and -middle occipital gyrus circuits during the POV phase. For the between-phase planned contrast, PDM group exhibited hypoconnectivity of the aIPS-bilateral posterior insula (DAN-pain matrix) and -right postcentral gyrus circuits (DAN-pain matrix) during the MENS phase as compared to the POV phase (Fig. 2B; Table 3). No significant between-phase difference was found in the CON group.

#### Correlation between FC and psychological measurements

**Figure 3:**
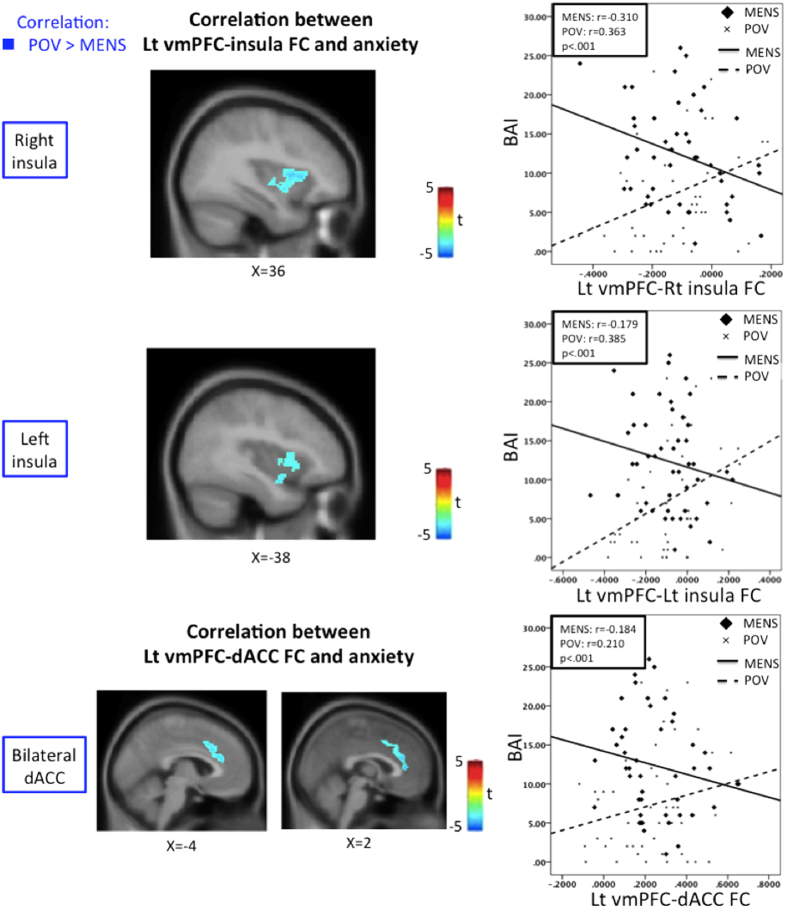
Correlation between FC and anxiety score inverses across phases in PDM. The Lt vmPFC-Rt insula, -Lt insula and -bilateral dorsal anterior cingulate cortex (dACC) FCs are positively correlated with BAI during the POV phase but negatively correlated with BAI during the MENS phase. Original FC values were used for the correlation to avoid artificial and inadvertent zeroing. Blue regions are associated with lower correlation with BAI during MENS phase. Significance thresholded at the uncorrected voxel level p = 0.005 followed by the FWE-corrected cluster level p = 0.05. BAI, Beck Anxiety Inventory; Lt, left; Rt, right.

In the PDM group, the FC of the left vmPFC-bilateral posterior-mid insula (DMN-pain matrix connectivity) and -dACC (DMN-pain matrix connectivity) were more correlated with BAI during the POV phase when compared to the MENS phase (Fig. 3; Table 4). The FCs of the left vmPFC-bilateral midcingulate cortex and -right middle frontal gyrus positively correlated with BAI, and the FC of the left vmPFC-bilateral declive negatively correlated with BAI during the POV phase. The FC of the right vmPFC-right inferior temporal gyrus and -left declive circuit negatively correlated with BAI during the POV phase. The FCs of the aIPS-bilateral rectal gyrus and -right cerebellum connectivity and BAI were significantly more correlated during the POV phase when compared with the MENS phase. The FC of the aIPS-bilateral rectal gyrus positively correlated with BAI and the FC of the aIPS-left precuneus negatively correlated with BAI during the POV phase. For brain-behavioral correlations, there was no significant correlation during the MENS phase (Table 4).

## Discussion

Using ReHo and ReHo-seeded FC analyses, the current study demonstrated *state*- and *trait-related* intra- and inter-regional FC alterations that collectively may result in subclinical central modulations of pain in otherwise healthy women with PDM. In our study, we observed a decreased ReHo, or a reduced local synchrony, in the vmPFC, which indicates increased intra-regional functional segregations in reaction to long-term menstrual pain. We also measured changes of inter-regional FC (both intrinsic and extrinsic network connectivity) in the PDM group, which may represent network reorganization in the context of strengthened DMN-ECN FC and attenuated DMN-SN FC when compared to the CON group. In addition, functional interaction between aIPS/SPL and pain-related regions (DAN-pain matrix connectivity) might be associated with a top-down regulation of pain transmission. Finally, we observed an inverse correlation between the vmPFC-posterior/mid insula FC (DMN-pain matrix connectivity) and anxiety scores across different phases. This suggests an adaptive neuroplasticity that is related to pain-primed mental state changes.

The vmPFC of PDM group manifested a relative lack of resilience (Fig. 1A) across the menstrual cycle as reflected in the significantly lower ReHo in the PDMs vs. CONs. The functional expression of DMN throughout the menstrual cycle differed between PDM and CON groups. The stereotyped desynchronized local activity (similar ReHo value across the menstrual cycle) in the PDMs indicates sustained and constrained neuroplasticity for divergent local functionalities or enhanced intra-regional functional segregation^31^. Being a part of the DMN and a functional hub of the brain, the vmPFC integrates and regulates conceptual information, including affective sensory, self-perception, social cognition, and emotion^10^,^33^. The vmPFC is also involved in the anticipatory coping mechanism^34^. It has also been suggested that the vmPFC and the linked nucleus accumbens constitute a system that mediates the effects of self-regulation on pain evaluation^14^,^35^. Of note, the DMN is a key network altered in chronic pain diseases^17^ and abnormal resting-state FC of it can be associated with pain intensity and rumination^36^,^37^,^38^. The ReHo reduction herein could possibly be attributed to various factors, such as the self-regulation of emotion, behavioral coping, appraisal/re-appraisal of self-relevant social information, and adaptation for pain modulation in relevant networks in response to PDM.

The functional expression and engagement of DAN throughout the menstrual cycle differed between PDM and CON groups, as reflected by the interaction effect of group and phase. The aIPS/SPL services attention shifting and saliently driven sensory discrimination and is a cardinal substrate of the DAN that participates in the top-down attention to pain^22^. Therefore, the relative lower ReHo of aIPS/SPL during the MENS phase vs. the POV phase in the PDM group (Figs 1A and 2A) might implicate an active disengagement of pain attention as part of salience modulation. The relative higher ReHo of the aIPS/SPL during the POV phase might connote an overt integration of the functionality for salience detection for the forthcoming stressful events.

Greater ReHo (MENS vs. POV) of the declive and the orbitofrontal cortex (OFC) in the PDM as compared to the CON group (Fig. 1A) could be adaptive to long-term menstrual pain. The OFC plays an important role in visceral sensory/nociceptive processing, and is activated during MENS phase of PDMs^6^. The OFC is crucial to adaptively cope with physiological challenges, regulate affective states and modulate pain^39^. Of note, we also found greater left vmPFC-SMA FC during MEMS (vs. POV phase) in PDM group as compared to CON group (Fig. 1B). The medial brain motor area has been suggested to be a visceromotor area and has underrecognized modulatory influence on visceral processes^40^. In addition, many chronic pain conditions are associated with increased DMN-SMN (including SMA) FC^17^,^20^. The group-phase interaction of vmPFC-SMA FC (DMN-SMN network) indicates that the cross-network communication is altered in the PDM group. Similar to our recent finding of periaqueductal gray matter (PAG) FC changes to the DMN^7^, the altered DMN-SMN network may be an ongoing representation of cumulative menstrual pain, which might be generated in part by FC dysfunction between medial brain motor regions and both DMN and PAG^40^.

The functional interplay between the three core networks might reorganize after stress events^41^. In the present study, we observed reduced connectivity in the vmPFC-dACC circuit (DMN-SN network) in the PDM group. The dACC is a critical neural substrate of the SN network^42^. Hong *et al.*^43^ suggested that females allocate more neural resources to interoceptive awareness. The authors demonstrated hypoconnectivity between the SN- and DMN-related regions in patients with irritable bowel syndrome (a chronic pain condition that is often comorbid with PDM). Such reduced DMN-SN FC can be adaptive for stress and anxiety^44^ and perhaps a reactive modulation to PDM. Conversely, we found hyperconnectivity in the vmPFC-VPL (DMN-ECN network) and vmPFC-DLPFC circuits (DMN-ECN network) in the PDM group. These two regions of the ECN are associated with pain attention and modulation^22^,^45^. The dynamic transition from the DMN-SN to DMN-ECN FC in individuals with PDM might implicate a shift from affective processing of pain salience to the cognitive modulation of long-term menstrual pain. DMN engagement is essential to an improved performance in self-related tasks^46^, including the reappraisal (involving both ECN and DMN^47^) of potentially harmful inputs that can intervene relatively early in the process of emotion-generation and the recruitment of executive cognitive control processes. This cross-network coordination plausibly implicates a functional plasticity and a coping mechanism underlying a normal protective adaptation in females with PDM as a result of repeated cyclic stress.

The right anterior insula and right inferior frontal gyrus exhibited *trait-related* decrease of FC with right vmPFC (Fig. S4, Table 2), and are involved in the SN and right-lateralized ventral attention network (VAN), respectively; possibly encoding the prolonged salience of pain^48^. The hypoconnectivity might connote a possible coping in active inhibition of interoceptive awareness for pain saliency and attention to pain. The hypoconnectivity of the aIPS-right postcentral gyrus and -bilateral posterior-mid insula FCs (DAN-pain matrix connectivity) can also be adaptive in patients with chronic PDM. The postcentral gyrus and posterior-mid insula service the discriminative dimension of pain experience^49^,^50^. The posterior insula integrates viscerosensory and motor processing, and participate in the interaction between motor cortical areas controlling painful body regions and distant non-motor cortical brain regions^51^. As a neural substrate of the DAN, the aIPS/SPL has an important role in top-down attention regulation^52^, which modulates pain perception^22^. Diverting attention from pain can decrease the activity of the ascending pain system^53^ and can be mechanistic for cognitive–behavioral therapy and meditation^54^. Grant *et al.*^55^ reported that meditators who are less sensitive to pain feature a decoupled FC between the dACC and the dorsolateral prefrontal cortex. This disengagement from higher-order evaluative processes has an important role in the regulation of pain and emotion as well as in cognitive control^53^.

As a part of the pain matrix^50^, the insula integrates interoception into the subjective social-emotion^56^ and has an important role in allocating attention and evaluating context^57^,^58^. Beside, dACC and mPFC are associated with interoceptive awareness, and play an important role in the appraisal and expression of fear and anxiety^10^. Increased DMN/mPFC-insula FC has been reported in several chronic pain disorders^18^,^37^. The alteration of mPFC-insula FC is also present in the unpleasant itch condition^59^. Chronic pain and itch may invoke guarding or scratching behaviors and elicit anxiety concomitantly. Hence, the mPFC-insula FC could be a state-specific biomarker for chronic pain perception^38^. This proposition has been corroborated by a study that found that opioids exert their analgesic efficacy by disrupting the vmPFC/rACC-insula FC^60^. State anxiety is positively correlated with DMN-insula FC in healthy subjects^61^. Although pain and anxiety are based on distinguishable neural mechanisms^62^, anxiety is often accompanied by menstrual pain^3^,^5^,^6^. In the present study, a trend of positive correlation between mPFC-posterior-mid insula FC (DMN-pain matrix connectivity) and anxiety was observed during the POV phase. This finding is consistent with the view that neuroticism can be a personality trait in patients with PDM^63^. The positive correlation shifted to a negative correlation when menstrual pain was present, thereby implicating a possible adaptation to pain-primed anxiety.

Pain is a subjective experience with wide individual differences. One possible explanation is that females with PDM have a propensity for anxiety that can be attributed to genetic factors, such as the *BDNF* Val66Met polymorphism^3^. *BDNF* genotypes may exhibit cognitive and neurobiological differences^64^. In addition, menstrual pain is accompanied by complex and subtle changes in emotion and cognition. The associated coping strategies and emotional and cognitive regulation vary across individuals. It can be heuristic to explore the interaction between the *BDNF* Val66Met polymorphism and its neuronal regulation to stress in individuals with PDM.

The neuroimaging is a sensitive measurement that can provide subclinical or preclinical evidence of significant alterations in brain function even without conspicuous behavioral manifestations^65^. Our results suggest that PDM subjects, after long-term cyclic menstrual pain, may develop neuroplasticity and brain reorganization as manifested by intra- and inter-regional FC changes (intrinsic and extrinsic network connectivity) of neural networks. The interoceptive awareness and switching roles of the SN network for pain saliency can be substituted by cognitive control function of the ECN network. Such resilient transition can be adaptive in long-term PDM subjects and may contribute to the understanding of system dynamics of functional pain connectome in other acute and chronic pain conditions^15^.

## Materials and Methods

### Subjects

The subjects of this study were a subset of the participants from our previous genetic association and behavioral studies of PDM who were eligible for neuroimaging studies^3^. Originally, 326 PDMs and 209 CONs were registered during August 2011 to March 2014 as intended for the integrated behavioral and multimodal imaging genetics studies (magnetic resonance imaging and magnetoencephalography). Two hundred and twenty-nine PDMs and 103 CONs were then excluded after rigorous screening according to the exclusion criteria and one or a combination of the following factors: an irregular menstrual cycle, a prolonged or shortened menstrual cycle, inconsistent pain intensity, pelvic abnormalities by ultrasonography as examined after entering the program, or being unwilling to complete the entire series of genetic, hormonal, behavioral, and multimodal neuroimaging studies (see also Lee *et al.*^3^). After entering the actual multimodal neuroimaging experiments, 11 PDMs and 3 CONs were excluded due to incidental brain findings (see also Li *et al.*^2^); 36 PDMs and 38 CONs excluded from subsequent analyses due to drop-out that ended in incomplete dataset; 2 PDMs and 10 CONs excluded owing to abnormal hormone level (possibly due to sampling and technical errors); and 2 PDMs and 6 CONs excluded owing to the head motion (>2 mm) or rotation (>2°) during the scan. Eventually, 46 PDMs and 49 CONs were eligible for neuroimaging analyses in this study (whole protocols completed), as also reported in our recently published functional connectivity study of PDM (see Wei *et al.*^7^).

The inclusion criteria for subjects with PDM included: 1) 20–30-year-old Taiwanese (Asian) females; 2) a regular menstrual cycle of approximately 27–32 days; 3) a history of menstrual pain longer than 6 months; 4) averaged menstrual pain under regular treatment with a rating at least higher than 4 on a verbal numerical scale (VNS, 0 = not at all, 10 = the worst imaginable pain) in the last 6 months; and 5) right-handedness, as confirmed by the Edinburgh Handedness Inventory. All subjects in the PDM group underwent pelvic ultrasonography to exclude secondary dysmenorrhea that was caused by organic pelvic diseases, such as endometriosis or adenomyosis. All participants were clinically examined and diagnosed in the gynecology clinic by the same gynecologist. The inclusion criteria for the healthy female control subjects were similar to those for the PDM group, with the exception that the members of the control group must have no pain during menses (VNS = 0). The exclusion criteria for all the participants were: 1) using oral contraceptives, hormonal supplements, Chinese herbal medicine, or any central-acting medication (e.g., opioid, anti-epileptics) within 6 months prior to the study; 2) pathological pituitary gland disease; 3) organic pelvic disease; 4) any psychiatric or neurological disorders; 5) brain trauma or brain surgery; 6) immediate plans for pregnancy or a positive pregnancy test; 7) a history of childbirth; and 8) having a metal/pacemaker implant, claustrophobia, or any contraindications to magnetic resonance imaging (MRI). No analgesics were used within 24 hours of the scans. The study was conducted in accordance with the Declaration of Helsinki and was approved by the Institutional Review Board of Taipei Veterans General Hospital. Written informed consents were obtained from all participants.

### Experimental design

MRI scanning was individually scheduled according to the commencement day of menstruation for each subject. Psychological assessments, blood samples for gonadal hormone assays and MRI images (T1 and resting-state fMRI images) were obtained at two time points during the menstrual cycle: the MENS phase (days 1–3 of the menstrual cycle) and the POV phase (days 12–16 of the menstrual cycle). Ovulation was confirmed using a urinary luteinizing hormone test (Han Chiun Proper LH Rapid Test).

### Psychological measurements

The patients in the PDM group completed the PCS and recounted their overall menstrual pain by using the McGill Pain Questionnaire during the inception interview. Then, during the MENS phase, subjects were assessed for their present experience of menstrual pain using the McGill Pain Questionnaire. All participants completed the BDI, BAI and STAI during the MENS and POV phases to assess their psychological status.

### Serum gonadal hormone measurements

The serum that was extracted during both phases was stored for batch analysis using commercialized assays (UniCel DxC 800 Synchron Clinical Systems, Beckman Coulter, Inc., Brea, CA). The total serum concentrations were assayed using a chemiluminescence immunoassay technique for estradiol and progesterone and a radioimmunoassay technique for the testosterone. As gonadal hormones may affect resting-state FC^66^, hormone fluctuations were regressed out as covariates of non-interest in the following image processing.

### Image acquisition

Resting-state functional MRI images were acquired using a 3.0 Tesla MRI scanner (Magnetom Trio Tim, Siemens, Erlangen, Germany) with a 12-channel head coil. High-resolution T1-weighted 3-dimensional structural images using a magnetization-prepared rapid-acquired gradient echo sequence (MPRAGE; [TR]/[TE] = 2530 ms/3.03 ms, flip angle = 70°, field-of-view = 224 × 256 × 192 mm^3^, in-plane matrix size = 224 × 256 × 192, in-plane resolution = 1 mm) and T2*-weighted gradient echo sequence ([TR]/[TE] = 2500 ms/30 ms, flip angle = 90°, field-of-view =220 × 220 × 136 mm^3^, in-plane matrix size = 64 × 64 × 40, in-plane resolution = 3.4 mm [round-out], and 204 volumes per run) were conducted to obtain high-resolution anatomical T1 images and functional MRI images. The first four functional scans of each resting-state functional MRI series were discarded for signal saturation and magnetic field stabilization. Participants remained awake during the scan and were instructed to maintain open eyes and relaxed, still heads without thinking about any topic in particular. Head cushions and earplugs were applied to reduce head motion and noise.

### Resting-state analysis

#### Regional homogeneity (ReHo) analysis

Preprocessing was performed by using the Data Processing Assistant for Resting-State fMRI (DPARSF) V2.3 Advanced Edition (State Key Laboratory of Cognitive Neuroscience and Learning, Beijing Normal University, China)^67^ with statistical parametrical mapping 8 (SPM 8; Wellcome Trust Center for Neuro-imaging, University College London, London, UK) in Matlab 2013b (The MathWorks, Inc., Natick, MA, USA). The preprocessing protocol was adapted from previous studies^68^. In brief, all functional images were slice-time corrected and realigned for head motion correction. Subjects with head motion of any volume more than 2 mm or 2° were excluded from further processing. Images were normalized using the SPM’s standard echo planar imaging template in Montreal Neurological Institute (MNI) space and re-sampled to an isotropic 2 mm^3^ voxel size. The resulting time series in each voxel was then linearly detrended and band-pass filtered (0.01–0.08 Hz) to extract the low-frequency oscillations. The following nuisance variables were regressed out: 1) the six head movement parameters computed based on rigid body translation and rotation during the realignment in SPM8; 2) the mean signal within the lateral ventricles for cerebral spinal fluid; and 3) the mean signal within a deep white matter region (centrum ovale). Brain activity was activated in clusters, or contiguous voxels, rather than in a few voxels. ReHo maps were generated by calculating the Kendall’s coefficient of concordance (KCC) of the time series between a given voxel with its nearest neighbors (26 voxels) in a voxel-wise manner. The ReHo maps were spatially smoothed using a 3D Gaussian kernel of 6 mm full-width at half-maximum (FWHM). For standardization purposes, each individual subject’s ReHo map was divided by its own global mean. The peaks of significant clusters were then selected as the ReHobased seeds.

#### Functional connectivity analysis of ReHo-based seeds

The preprocessing of the functional connectivity analysis protocol was as same as in the ReHo analysis, except that the images were 1) coregistered to individuals’ anatomical image and then normalized to the standard T1 MNI template; 2) spatially smoothed using a 3D Gaussian kernel of 8 mm FWHM; and 3) treated with the global mean signal for additional nuisance variables. We performed global signal regression because it has been shown to maximize the specificity of positive resting-state correlations when using real and simulated data^69^. However, the neuroscientific interpretation of anti-correlation has been challenged^70^, and global signal regression may cause a negative shift in the distribution of correlations^71^,^72^; therefore, we implemented a mask and addressed positive connectivity only^7^. The seeded regions that were selected for FC analysis were those wherein the ReHo significantly differed in the between-group and between-phase comparisons. The mean time series activity was extracted within the 3mm radius spherical ReHoseeded regions. There is no strict universal standard or any fixed parameter for the seed definition in various modalities of neuroimaging. We had used 3 mm as the radius for the seeds in our published functional studies^7^,^73^. Three-mm was chosen out of the concern to avoid additional partial volume effect since our original fMRI resolution is 3.4 × 3.4 × 3.4 mm and the cortical thickness as reported is about 2 to 4 mm across the brain^74^. The individual FC maps were computed by the Pearson’s correlation coefficient (r) between the seeds and the related brain regions. After calculating the correlation between the reference time course and the time course of each voxel in the brain, r values were converted into z-values using Fisher’s r-to-z transformation to normalize the distribution. Finally, we only used positive FC maps in subsequent analyses to minimize the possible influences of artifacts of anticorrelations.

#### Network assignments for the altered ReHo and FC regions

For the network assignments for the significantly altered ReHo and FC regions, we conducted the inverse validation by means of FC analyses, using peaks of significant clusters as the seeds. The FC was considered significant if family-wise error level was p < 0.05 at voxel level. The FC map of each seed was visually compared with the FC map of the key brain systems as published previously^75^. Basically, all the reported network assignments were affirmed by this procedure.

### Statistical analyses

#### Demographic and psychological data

SPSS Statistics 20.0 (SPSS Inc., Chicago, IL) was used for all analyses. Two-sample t-tests were conducted for the between-group (PDM vs. CON) differences in demographic characteristics and Edinburgh Handedness Inventory scores. To test the group-phase interactions, we used mixed design two-way ANOVA. The significance was thresholded at p = 0.05. For more comprehensive statistical analyses and results, please refer to our previous paper by Lee *et al.*^3^.

#### Image data: ReHo and ReHo-seeded FC

For the interaction effects of group and phase, we compared the phase difference (MENS phase vs. POV phase) of mean ReHo values and ReHo-seeded FCs between the PDM and CON groups by the factorial design module of SPM8. To elucidate the respective *trait*- and *state*-related neuroplasticity, we focused on the planned contrasts of between-group differences of respective phase and between-phase differences of respective group rather than the main effects for ReHo values and ReHo-seeded FCs^5^,^7^. The planned between-group and between-phase comparisons of imaging data were conducted using 2-sample and paired *t* test, respectively. Main effects analyses were also conducted for reference only. Three gonadal hormone levels were treated as non-interest covariates. Significance was thresholded at the uncorrected voxel level p = 0.005 followed by the FWE-corrected cluster level p = 0.05.

#### Image data: brain-behavior correlation

Since studies have reported that state anxiety correlate with mPFC-insula FC^61^, we also applied regression analyses between ReHo-seeded FC and BAI for the PDM group. Three gonadal hormone levels were treated as non-interest covariates. Significance was thresholded at the uncorrected voxel level p = 0.005 followed by the FWE-corrected cluster level p = 0.05.

## Additional Information

**How to cite this article**: Wu, T.-H. *et al.* Dynamic Changes of Functional Pain Connectome in Women with Primary Dysmenorrhea. *Sci. Rep.* **6**, 24543; doi: 10.1038/srep24543 (2016).

## Supplementary Material

Supplementary Information

## Figures and Tables

**Table 1:** The main effect of group and phase, interaction effect, between-group differences, and within-group differences in regional homogeneity.

Contrast	Region	BA	Size	*t*_score_	Coordinate		
					x	y	z
Main effects of group (PDM vs. CON)							
PDM > CON	NS						
CON > PDM	NS						
Main effects of phase (MENS vs. POV)							
MENS > POV	NS						
POV > MENS	NS						
Interaction							
PDM_Δ(MENS-POV)_ > CON_Δ(MENS-POV)_	declive	–	415	4.62	−22	−68	−26
	OFC	10	512	3.94	38	52	−12
CON_Δ(MENS-POV)_ > PDM_Δ(MENS-POV)_	aIPS/SPL	40	429	4.58	−42	−38	52
Between-group planned contrast							
MENS: PDM > CON	NS						
MENS: CON > PDM	NS						
POV: PDM > CON	NS						
POV: CON > PDM	vmPFC	10	377	3.89	6	62	22
				3.6	0	66	12
				3.51	−6	50	4
Between-phase planned contrast							
PDM: MENS > POV	NS						
PDM: POV > MENS	aIPS/SPL/VPL	40	520	5.11	−42	−36	48
				3.5	−30	−26	44
				3.39	−36	−46	56
CON: MENS > POV	NS						
CON: POV > MENS	declive	−	409	5.22	−22	−68	−26

Peak coordinates refer to Montreal Neurological Institute space. Significance was set at the uncorrected voxel level p = 0.005 followed by the FWE-corrected cluster level p = 0.05. BA, Brodmann area; OFC, orbitofrontal cortex; vmPFC, ventromedial prefrontal cortex; VPL, ventral parietal lobe; aIPS, anterior part of the intraparietal sulcus; SPL, superior parietal lobe; MENS, menstruation phase; POV, periovulatory phase; PDM, primary dysmenorrhea group; CON, control group; NS, non-significant.

**Table 2:** Significant interaction effect, between-group and within-group differences in ReHo-seed (vmPFC) functional connectivity analyses.

Contrast	Region	BA	Size	*t*_score_	Coordinate		
					x	y	z
left side ReHo-seed (−6, 50, 4)							
Interaction							
PDM_Δ(MENS-POV)_ > CON_Δ(MENS-POV)_	SMA/MCC	6	539	3.95	2	−6	50
Between-group planned contrast							
MENS: PDM > CON	SFG	6	388	3.97	−14	24	58
	dmPFC	9	384	3.94	−10	48	28
MENS: CON > PDM	dACC	32	374	4.2	2	20	32
POV: PDM > CON	dmPFC/DLPFC	8	952	4.24	−12	30	40
	VPL/angular	39	372	3.85	−42	−68	48
POV: CON > PDM	pACC	24	823	4.28	0	38	10
					2	22	28^#^
Between-phase planned contrast							
CON: POV > MENS	SMA	6	580	4.46	−12	−12	54
right side ReHo-seed (6, 62, 22)							
Between-group planned contrast							
POV: CON > PDM	IFG	–	335	5.06	46	20	6
	pACC	32	691	3.99	4	40	8
Between-phase planned contrast							
PDM: POV > MENS	pons	–	325	4.58	12	−38	−34

Peak coordinates refer to Montreal Neurological Institute space. Significance thresholded at the uncorrected voxel level p = 0.005 followed by the FWE-corrected cluster level p = 0.05.

SMA, supplementary motor area; MCC, midcingulate cortex; SFG, superior frontal gyrus; dmPFC, dorsomedial prefrontal cortex; dACC, dorsal anterior cingulate cortex; DLPFC, dorsolateral prefrontal cortex; VPL, ventral parietal lobe; pACC, pregenual anterior cingulate cortex; IFG, inferior frontal gyrus (see Table 1 for other abbreviation details) ^#^the second highest peak, almost the same location with the highest peak in the MENS phase.

**Table 3:** Significant between-group and within-group differences in ReHo-seed (aIPS/SPL) functional connectivity analyses.

Contrast	Region	BA	Size	*t*_score_	Coordinate		
					x	y	z
Between-group planned contrast							
MENS: PDM > CON	MTG	39	323	3.71	40	−60	20
MENS: CON > PDM	cerebellum anterior lobe	31	378	4.34	6	−48	−34
POV: PDM > CON	STG/MTG	22	996	4.54	56	−56	6
	MOG	19	469	4.27	−34	−80	−2
Between-phase planned contrast							
PDM: POV > MENS	claustrum	–	351	4.01	−36	−8	0
	supramarginal	40	498	4.35	54	−26	18
	insula	13	434	4.16	42	2	6

Peak coordinates refer to Montreal Neurological Institute space. Significance was set at the uncorrected voxel level p = 0.005 followed by the FWE-corrected cluster level p = 0.05. MTG, middle temporal gyrus; STG, superior temporal gyrus; MOG, middle occipital gyrus.

**Table 4:** ReHo-seeded functional connectivity significantly covarying with the Beck Anxiety Index (BAI).

ReHo-seed	Positive correlation							Negative correlation						
	Region	BA	Size	*t*_score_	Coordinate			Region	BA	Size	*t*_score_	Coordinate		
*phase contrast*					X	Y	Z					X	Y	Z
left vmPFC (−6, 50, 4)														
*POV*														
	MFG	10	388	4.69	28	48	12	declive	–	265	3.99	−12	−68	−22
	MCC	24	354	4.04	2	2	38							
*MENS* > *POV*														
	NS							insula/dACC	–	1292	4.84	36	−10	−2
											4.04	−6	22	26
											3.82	2	36	8
								insula	–	618	4.26	−28	4	14
right vmPFC (6, 62, 22)														
*POV*														
	NS							ITG	21	273	4.34	58	−10	−18
								declive	–	431	3.9	−10	−74	−28
aIPS/SPL (−42, −36, 48)														
*POV*														
	rectal gyrus	11	536	5.09	−4	28	−26	precuneus/postcentral gyrus	5/7	874	4.76	−6	−50	66
*MENS* > *POV*														
	NS							rectal gyrus	11	803	5	4	18	−26
								cerebellum	–	351	4.59	50	−70	−36

Peak coordinates refer to Montreal Neurological Institute space. Significance was set at the uncorrected voxel level p = 0.005 followed by the FWE-corrected cluster level p = 0.05.

MFG, middle frontal gyrus; MCC, midcingulate cortex; ITG, inferior temporal gyrus; dACC, dorsal anterior cingulate cortex; NS, non-significant. (see Table 1 for other abbreviation details).

## References

[b1] Berkley K. J. Primary Dysmenorrhea: An Urgent Mandate. Pain: Clinical Updates 21(3), 1–8 (2013).

[b2] Li W. C. *et al.* High prevalence of incidental brain findings in primary dysmenorrhoea. Eur J Pain 19, 1071–1074 (2015). 25487523 10.1002/ejp.639

[b3] Lee L. C. *et al.* Association of brain-derived neurotrophic factor gene Val66Met polymorphism with primary dysmenorrhea. Plos One 9, e112766 (2014). 25383981 10.1371/journal.pone.0112766 PMC4226574

[b4] Tu C. H. *et al.* Brain morphological changes associated with cyclic menstrual pain. Pain 150, 462–468 (2010). 20705214 10.1016/j.pain.2010.05.026

[b5] Tu C. H. *et al.* Menstrual pain is associated with rapid structural alterations in the brain. Pain 154, 1718–1724 (2013). 23693160 10.1016/j.pain.2013.05.022

[b6] Tu C. H. *et al.* Abnormal cerebral metabolism during menstrual pain in primary dysmenorrhea. NeuroImage 47, 28–35 (2009). 19362153 10.1016/j.neuroimage.2009.03.080

[b7] Wei S. Y. *et al.* Changes in functional connectivity of pain modulatory systems in women with primary dysmenorrhea. Pain 157, 92–102 (2016). 26307856 10.1097/j.pain.0000000000000340

[b8] Vincent K. *et al.* Dysmenorrhoea is associated with central changes in otherwise healthy women. Pain 152, 1966–1975 (2011). 21524851 10.1016/j.pain.2011.03.029

[b9] Melzack R. Phantom limbs and the concept of a neuromatrix. Trends Neurosci. 13, 88–92 (1990). 1691874 10.1016/0166-2236(90)90179-e

[b10] Etkin A., Egner T. & Kalisch R. Emotional processing in anterior cingulate and medial prefrontal cortex. Trends Cogn Sci. 15, 85–93 (2011). 21167765 10.1016/j.tics.2010.11.004 PMC3035157

[b11] Rudorf S. & Hare T. A. Interactions between dorsolateral and ventromedial prefrontal cortex underlie context-dependent stimulus valuation in goal-directed choice. J Neurosci. 34, 15988–15996 (2014). 25429140 10.1523/JNEUROSCI.3192-14.2014 PMC6608472

[b12] Maren S., Phan K. L. & Liberzon I. The contextual brain: implications for fear conditioning, extinction and psychopathology. Nat Rev Neurosci. 14, 417–428 (2013). 23635870 10.1038/nrn3492 PMC5072129

[b13] Tracey I. Getting the pain you expect: mechanisms of placebo, nocebo and reappraisal effects in humans. Nat Med. 16, 1277–1283 (2010). 20948533 10.1038/nm.2229

[b14] Lee M. *et al.* Activation of corticostriatal circuitry relieves chronic neuropathic pain. J Neurosci. 35, 5247–5259 (2015). 25834050 10.1523/JNEUROSCI.3494-14.2015 PMC4380998

[b15] Kucyi A. & Davis K. D. The dynamic pain connectome. Trends Neurosci. 38, 86–95 (2015). 25541287 10.1016/j.tins.2014.11.006

[b16] Ceko M. *et al.* Partial recovery of abnormal insula and dorsolateral prefrontal connectivity to cognitive networks in chronic low back pain after treatment. Hum Brain Mapp. 36, 2075–2092 (2015). 25648842 10.1002/hbm.22757 PMC6869701

[b17] Hemington K. S., Wu Q., Kucyi A., Inman R. D. & Davis K. D. Abnormal cross-network functional connectivity in chronic pain and its association with clinical symptoms. Brain Struct Funct. (2015) (doi:10. 1007/s00429-015-1161-1). 10.1007/s00429-015-1161-1 26669874

[b18] Baliki M. N., Mansour A. R., Baria A. T. & Apkarian A. V. Functional reorganization of the default mode network across chronic pain conditions. Plos One 9, e106133 (2014). 25180885 10.1371/journal.pone.0106133 PMC4152156

[b19] Kucyi A., Salomons T. V. & Davis K. D. Mind wandering away from pain dynamically engages antinociceptive and default mode brain networks. Proc Natl Acad Sci. USA 110, 18692–18697 (2013). 24167282 10.1073/pnas.1312902110 PMC3832014

[b20] Makin T. R. *et al.* Network-level reorganisation of functional connectivity following arm amputation. NeuroImage 114, 217–225 (2015). 25776216 10.1016/j.neuroimage.2015.02.067 PMC4461307

[b21] McKlveen J. M., Myers B. & Herman J. P. The medial prefrontal cortex: coordinator of autonomic, neuroendocrine and behavioural responses to stress. J Neuroendocrinol. 27, 446–456 (2015). 25737097 10.1111/jne.12272 PMC4580281

[b22] Bushnell M. C., Ceko M. & Low L. A. Cognitive and emotional control of pain and its disruption in chronic pain. Nat Rev Neurosci. 14, 502–511 (2013). 23719569 10.1038/nrn3516 PMC4465351

[b23] Seminowicz D. A. & Davis K. D. Pain enhances functional connectivity of a brain network evoked by performance of a cognitive task. J Neurophysiol 97, 3651–3659 (2007). 17314240 10.1152/jn.01210.2006

[b24] Seidel E. M. *et al.* Uncertainty during pain anticipation: the adaptive value of preparatory processes. Hum Brain Mapp. 36, 744–755 (2015). 25324216 10.1002/hbm.22661 PMC6869185

[b25] Seeley W. W. *et al.* Dissociable intrinsic connectivity networks for salience processing and executive control. J Neurosci. 27, 2349–2356 (2007). 17329432 10.1523/JNEUROSCI.5587-06.2007 PMC2680293

[b26] Spreng R. N., Sepulcre J., Turner G. R., Stevens W. D. & Schacter D. L. Intrinsic architecture underlying the relations among the default, dorsal attention, and frontoparietal control networks of the human brain. J Cogn Neurosci. 25, 74–86 (2013). 22905821 10.1162/jocn_a_00281 PMC3816715

[b27] Whitburn L. Y., Jones L. E., Davey M. A. & Small R. Women’s experiences of labour pain and the role of the mind: an exploratory study. Midwifery 30, 1029–1035 (2014). 24820004 10.1016/j.midw.2014.04.005

[b28] Gagua T., Tkeshelashvili B., Gagua D. & McHedlishvili N. Assessment of anxiety and depression in adolescents with primary dysmenorrhea: a case-control study. J Pediatr Adolesc Gynecol. 26, 350–354 (2013). 24075089 10.1016/j.jpag.2013.06.018

[b29] Zang Y., Jiang T., Lu Y., He Y. & Tian L. Regional homogeneity approach to fMRI data analysis. NeuroImage 22, 394–400 (2004). 15110032 10.1016/j.neuroimage.2003.12.030

[b30] Zhong Y. *et al.* Altered regional synchronization in epileptic patients with generalized tonic-clonic seizures. Epilepsy Res. 97, 83–91 (2011). 21856123 10.1016/j.eplepsyres.2011.07.007

[b31] Iwabuchi S. J. *et al.* Localized connectivity in depression: A meta-analysis of resting state functional imaging studies. Neurosci Biobehav Rev. 51, 77–86 (2015). 25597656 10.1016/j.neubiorev.2015.01.006

[b32] Yan F. X. *et al.* Resting-state functional magnetic resonance imaging analysis with seed definition constrained by regional homogeneity. Brain Connect. 3, 438–449 (2013). 23802999 10.1089/brain.2013.0164

[b33] Roy M., Shohamy D. & Wager T. D. Ventromedial prefrontal-subcortical systems and the generation of affective meaning. Trends Cogn sci. 16, 147–156 (2012). 22310704 10.1016/j.tics.2012.01.005 PMC3318966

[b34] Hsieh J. C., Stone-Elander S. & Ingvar M. Anticipatory coping of pain expressed in the human anterior cingulate cortex: a positron emission tomography study. Neurosci Lett. 262, 61–64 (1999). 10076873 10.1016/s0304-3940(99)00060-9

[b35] Woo C. W., Roy M., Buhle J. T. & Wager T. D. Distinct brain systems mediate the effects of nociceptive input and self-regulation on pain. Plos Biol. 13, e1002036 (2015). 25562688 10.1371/journal.pbio.1002036 PMC4285399

[b36] Kucyi A. *et al.* Enhanced medial prefrontal-default mode network functional connectivity in chronic pain and its association with pain rumination. J Neurosci. 34, 3969–3975 (2014). 24623774 10.1523/JNEUROSCI.5055-13.2014 PMC6705280

[b37] Napadow V. *et al.* Intrinsic brain connectivity in fibromyalgia is associated with chronic pain intensity. Arthritis Rheum. 62, 2545–2555 (2010). 20506181 10.1002/art.27497 PMC2921024

[b38] Loggia M. L. *et al.* Default mode network connectivity encodes clinical pain: an arterial spin labeling study. Pain 154, 24–33 (2013). 23111164 10.1016/j.pain.2012.07.029 PMC3534957

[b39] Garcia-Larrea L. & Peyron R. Pain matrices and neuropathic pain matrices: a review. Pain 154, Suppl 1, S29–43 (2013). 24021862 10.1016/j.pain.2013.09.001

[b40] Kutch J. J. & Tu F. F. Altered brain connectivity in dysmenorrhea: pain modulation and the motor cortex. Pain 157, 5–6 (2016). 26683107 10.1097/j.pain.0000000000000364 PMC4941100

[b41] Bosch O. G. *et al.* Sleep deprivation increases dorsal nexus connectivity to the dorsolateral prefrontal cortex in humans. Proc Natl Acad Sci. USA 110, 19597–19602 (2013). 24218598 10.1073/pnas.1317010110 PMC3845164

[b42] Hermans E. J., Henckens M. J., Joels M. & Fernandez G. Dynamic adaptation of large-scale brain networks in response to acute stressors. Trends Neurosci. 37, 304–314 (2014). 24766931 10.1016/j.tins.2014.03.006

[b43] Hong J. Y. *et al.* Sex and disease-related alterations of anterior insula functional connectivity in chronic abdominal pain. J Neurosci. 34, 14252–14259 (2014). 25339739 10.1523/JNEUROSCI.1683-14.2014 PMC4205551

[b44] Pannekoek J. N. *et al.* Resting-state functional connectivity abnormalities in limbic and salience networks in social anxiety disorder without comorbidity. Eur Neuropsychopharmacol. 23, 186–195 (2013). 22749355 10.1016/j.euroneuro.2012.04.018

[b45] Lorenz J., Minoshima S. & Casey K. L. Keeping pain out of mind: the role of the dorsolateral prefrontal cortex in pain modulation. Brain 126, 1079–1091 (2003). 12690048 10.1093/brain/awg102

[b46] Spreng R. N. *et al.* Goal-congruent default network activity facilitates cognitive control. J Neurosci. 34, 14108–14114 (2014). 25319706 10.1523/JNEUROSCI.2815-14.2014 PMC4198547

[b47] Ochsner K. N., Silvers J. A. & Buhle J. T. Functional imaging studies of emotion regulation: a synthetic review and evolving model of the cognitive control of emotion. Ann N Y Acad sci. 1251, E1–24 (2012). 23025352 10.1111/j.1749-6632.2012.06751.x PMC4133790

[b48] Downar J., Mikulis D. J. & Davis K. D. Neural correlates of the prolonged salience of painful stimulation. NeuroImage 20, 1540–1551 (2003). 14642466 10.1016/s1053-8119(03)00407-5

[b49] Apkarian A. V., Bushnell M. C., Treede R. D. & Zubieta J. K. Human brain mechanisms of pain perception and regulation in health and disease. Eur J Pain 9, 463–484 (2005). 15979027 10.1016/j.ejpain.2004.11.001

[b50] Wiech K., Jbabdi S., Lin C. S., Andersson J. & Tracey I. Differential structural and resting state connectivity between insular subdivisions and other pain-related brain regions. Pain 155, 2047–2055 (2014). 25047781 10.1016/j.pain.2014.07.009 PMC4220010

[b51] Kutch J. J. *et al.* Altered resting state neuromotor connectivity in men with chronic prostatitis/chronic pelvic pain syndrome: A MAPP: Research Network Neuroimaging Study. NeuroImage Clin 8, 493–502 (2015). 26106574 10.1016/j.nicl.2015.05.013 PMC4474411

[b52] Humphreys G. F. & Lambon Ralph M. A. Fusion and Fission of Cognitive Functions in the Human Parietal Cortex. Cereb Cortex 25, 3547–3560 (2015). 25205661 10.1093/cercor/bhu198 PMC4585503

[b53] Wiech K., Ploner M. & Tracey I. Neurocognitive aspects of pain perception. Trends Cogn sci. 12, 306–313 (2008). 18606561 10.1016/j.tics.2008.05.005

[b54] Salomons T. V., Moayedi M., Erpelding N. & Davis K. D. A brief cognitive-behavioural intervention for pain reduces secondary hyperalgesia. Pain 155, 1446–1452 (2014). 24569149 10.1016/j.pain.2014.02.012

[b55] Grant J. A., Courtemanche J. & Rainville P. A non-elaborative mental stance and decoupling of executive and pain-related cortices predicts low pain sensitivity in Zen meditators. Pain 152, 150–156 (2011). 21055874 10.1016/j.pain.2010.10.006

[b56] Craig A. D. Interoception: the sense of the physiological condition of the body. Curr Opin Neurobiol. 13, 500–505 (2003). 12965300 10.1016/s0959-4388(03)00090-4

[b57] Berntson G. G. *et al.* The insula and evaluative processes. Psychol sci. 22, 80–86 (2011). 21148459 10.1177/0956797610391097 PMC3261800

[b58] Wiech K. *et al.* Anterior insula integrates information about salience into perceptual decisions about pain. J Neurosci. 30, 16324–16331 (2010). 21123578 10.1523/JNEUROSCI.2087-10.2010 PMC6634837

[b59] Desbordes G. *et al.* Evoked itch perception is associated with changes in functional brain connectivity. NeuroImage Clin 7, 213–221 (2015). 25610783 10.1016/j.nicl.2014.12.002 PMC4300003

[b60] Gorka S. M., Fitzgerald D. A., de Wit H., Angstadt M. & Phan K. L. Opioid modulation of resting-state anterior cingulate cortex functional connectivity. J Psychopharmacol. 28, 1115–1124 (2014). 25237122 10.1177/0269881114548436 PMC5613932

[b61] Dennis E. L., Gotlib I. H., Thompson P. M. & Thomason M. E. Anxiety modulates insula recruitment in resting-state functional magnetic resonance imaging in youth and adults. Brain Connect 1, 245–254 (2011). 22433052 10.1089/brain.2011.0030 PMC3621677

[b62] Wiech K. *et al.* Dissociable neural mechanisms underlying the modulation of pain and anxiety? An FMRI pilot study. Plos One 9, e110654 (2014). 25502237 10.1371/journal.pone.0110654 PMC4266493

[b63] Fontana D. & Rees V. Primary dysmenorrhea, educational performance, and cognitive and affective variables in adolescent schoolgirls. Br J Educ Psychol. 52, 199–204 (1982). 7138775 10.1111/j.2044-8279.1982.tb00826.x

[b64] Pecina M. *et al.* Valence-specific effects of BDNF Val66Met polymorphism on dopaminergic stress and reward processing in humans. J Neurosci. 34, 5874–5881 (2014). 24760847 10.1523/JNEUROSCI.2152-13.2014 PMC3996214

[b65] Lui S. *et al.* High-field MRI reveals an acute impact on brain function in survivors of the magnitude 8.0 earthquake in China. Proc Natl Acad Sci. USA 106, 15412–15417 (2009). 19720989 10.1073/pnas.0812751106 PMC2735557

[b66] Petersen N., Kilpatrick L. A., Goharzad A. & Cahill L. Oral contraceptive pill use and menstrual cycle phase are associated with altered resting state functional connectivity. NeuroImage 90, 24–32 (2014). 24365676 10.1016/j.neuroimage.2013.12.016 PMC4113343

[b67] Chao-Gan Y. & Yu-Feng Z. DPARSF: A MATLAB Toolbox for “Pipeline” Data Analysis of Resting-State fMRI. Front Syst Neurosci. 4, 13 (2010). 20577591 10.3389/fnsys.2010.00013 PMC2889691

[b68] Hu S. *et al.* Association of cerebral networks in resting state with sexual preference of homosexual men: a study of regional homogeneity and functional connectivity. Plos One 8, e59426 (2013). 23555670 10.1371/journal.pone.0059426 PMC3605412

[b69] Weissenbacher A. *et al.* Correlations and anticorrelations in resting-state functional connectivity MRI: a quantitative comparison of preprocessing strategies. NeuroImage 47, 1408–1416 (2009). 19442749 10.1016/j.neuroimage.2009.05.005

[b70] Buckner R. L., Krienen F. M. & Yeo B. T. Opportunities and limitations of intrinsic functional connectivity MRI. Nat Neurosci. 16, 832–837 (2013). 23799476 10.1038/nn.3423

[b71] Fox M. D., Zhang D., Snyder A. Z. & Raichle M. E. The global signal and observed anticorrelated resting state brain networks. J Neurophysiol. 101, 3270–3283 (2009). 19339462 10.1152/jn.90777.2008 PMC2694109

[b72] Murphy K., Birn R. M., Handwerker D. A., Jones T. B. & Bandettini P. A. The impact of global signal regression on resting state correlations: are anti-correlated networks introduced? NeuroImage 44, 893–905 (2009). 18976716 10.1016/j.neuroimage.2008.09.036 PMC2750906

[b73] Ku H. L. *et al.* Brain signature characterizing the body-brain-mind axis of transsexuals. Plos One 8, e70808 (2013). 23923023 10.1371/journal.pone.0070808 PMC3724787

[b74] Fischl B. & Dale A. M. Measuring the thickness of the human cerebral cortex from magnetic resonance images. Proc Natl Acad sci. USA 97, 11050–11055 (2000). 10984517 10.1073/pnas.200033797 PMC27146

[b75] Raichle M. E. The restless brain. Brain Connect 1, 3–12 (2011). 22432951 10.1089/brain.2011.0019 PMC3621343

