# Supplementary material for: Dynamic Changes of Functional Pain Connectome in Women with Primary Dysmenorrhea
Source: Sci Rep. 2016 Apr 19;6:24543. doi: 10.1038/srep24543 (PMC4835697; doi:10.1038/srep24543)
Supplement: Supplementary Information [file srep24543-s1.pdf]

Dynamic Changes of Functional Pain Connectome in Women with Primary Dysmenorrhea

Ting-Hsuan Wu, Cheng-Hao Tu, Hsiang-Tai Chao, Wei-Chi Li, Intan Low, Chih-Ying Chuang, Tzu-Chen Yeh, Chou-Ming Cheng, Chih-Che Chou, Li-Fen Chen, and Jen-Chuen Hsieh

Supplementary Table S1. Demographic data, psychological/behavioral assessments and baseline information

	PDM	Control	Between-group	main effect		interaction
	(n = 46)	(n = 49)	p value (2-tailed)	group	phase	(group*phase)
Age, year	23.33±2.42	23.80±2.47	0.351	NA	NA	NA
Age at menarche	11.93±1.32	12.31±1.08	0.14	NA	NA	NA
Years of menstruating	11.39±2.84	11.49±2.86	0.867	NA	NA	NA
Days of one menstrual cycle	29.50±1.28	29.52±1.19	0.936	NA	NA	NA
Menstrual pain experience						
Pain history, year	9.22±2.85	NA	NA	NA	NA	NA
Absenteeism, %	65.2	NA	NA	NA	NA	NA
Drug taken, %	41.3	NA	NA	NA	NA	NA
Present PRI scores (range, 0-78)	30.11±11.99	NA	NA	NA	NA	NA
Sensory (range, 0-42)	16.54±6.01	NA	NA	NA	NA	NA
Affective (range, 0-14)	3.91±2.85	NA	NA	NA	NA	NA
Evaluation (range, 0-5)	2.54±2.02	NA	NA	NA	NA	NA
Miscellaneous (range, 0-17)	7.11±3.62	NA	NA	NA	NA	NA
Present PPI scores (range, 0-5)	2.72±0.96	NA	NA	NA	NA	NA
Edinburgh Handedness Inventory	82.96±16.05	80.20±20.44	0.467	NA	NA	NA
PCS total scores (range, 0-52)						
MENS phase	21.89±11.52	5.31±6.44	NA	<.001	.074	.017
POV phase	18.70±10.60	5.78±7.80	NA			
PCS: Pain rumination (range, 0-16)						
MENS phase	8.26±4.17	2.08±2.66	NA	<.001	.114	.214
POV phase	7.41±4.26	1.98±3.00	NA			
PCS: Pain helplessness (range, 0-24)						
MENS phase	10.22±5.80	2.22±2.84	NA	<.001	.057	.004
POV phase	8.22±5.06	2.63±3.72	NA			
PCS: Pain magnification (range, 0-12)						
MENS phase	3.41±2.46	1.00±1.56	NA	<.001	.609	.158
POV phase	3.07±2.49	1.16±1.70	NA			

Beck Anxiety Index (range, 0-63)						
MENS phase	12.35±6.46	2.42±2.77$	NA	<.001	<.001	<.001
POV phase	7.30±6.61	2.90±3.46	NA			
State-Trait Anxiety Inventory: State (range, 20-80)						
MENS phase	43.00±8.78	34.59±6.36	NA	<.001	<.001	<.001
POV phase	36.59±6.32	34.41±7.00	NA			
State-Trait Anxiety Inventory: Trait (range, 20-80)						
MENS phase	44.00±9.00	38.42±7.50$	NA	.005	.221	.092
POV phase	42.30±8.95	38.45±7.48	NA			
Beck Depression Index (range, 0-63)						
MENS phase	11.89±8.67	3.90±4.70	NA	<.001	<.001	<.001
POV phase	6.20±7.22	4.51±5.96	NA			

The data are presented as the means±SD.

$ Forty-eight subjects were included in the statistic because of data loss.

PDM, primary dysmenorrhea group; PCS, Pain catastrophizing scale; PRI, Pain Rating Index; PPI, Present Pain Intensity; NA, not available.

Supplementary Table S2. Between group comparisons of serum hormone levels

Hormone	Phase	PDM (n = 46)	Control (n = 49)	p value (two-tailed)
**Estradiol**				
	MENS	33.90±18.02	35.39±18.14	0.692
	POV	155.96±109.03	149.14±126.03	0.779
**Progesterone**				
	MENS	0.49±0.39	0.71±1.73	0.412
	POV	0.81±1.16	1.40±3.07	0.221
**Testosterone**				
	MENS	0.41±0.24	0.40±0.24	0.917
	POV	0.55±0.32	0.47±0.22	0.147

The data are presented as the means±SD.

PDM, primary dysmenorrhea; MENS, menstrual phase; POV, periovulatory phase.

Supplementary Table S3. The main effects of group and phase, interaction effect, between-group differences, and within-group differences in ReHo-seed (vmPFC) functional connectivity analyses

contrast	region	BA	size	t score	coordinate		
					x	y	z
**left side ReHo-seed (-6, 50, 4)**							
**Main effects of group (PDM vs. CON)**							
PDM>CON	SFG	6	420	4.63	-14	22	58
CON>PDM	NS						
**Main effects of phase (MENS vs. POV)**							
MENS>POV	NS						
POV>MENS	NS						
**Interaction (group*phase)**							
$PDM_{\Delta(MENS\text{-}POV)}$ > $CON_{\Delta(MENS\text{-}POV)}$	SMA/MCC	6	539	3.95	2	-6	50
$CON_{\Delta(MENS\text{-}POV)}$ > $PDM_{\Delta(MENS\text{-}POV)}$	NS						
**Between-group planned contrast**							
MENS: PDM>CON	SFG	6	388	3.97	-14	24	58
	dmPFC	9	384	3.94	-10	48	28
MENS: CON>PDM	dACC	32	374	4.2	2	20	32
POV: PDM>CON	dmPFC/DLPFC	8	952	4.24	-12	30	40
	VPL/angular	39	372	3.85	-42	-68	48
POV: CON>PDM	pACC	24	823	4.28	0	38	10
					2	22	28[#]
**Between-phase planned contrast**							
PDM: MENS>POV	NS						
PDM: POV>MENS	NS						
CON: MENS>POV	NS						
CON: POV>MENS	SMA	6	580	4.46	-12	-12	54
**right side ReHo-seed (6, 62, 22)**							
**Main effects of group (PDM vs. CON)**							
PDM>CON	NS						
CON>PDM	IFG/insula	13	460	4.63	40	14	16
	pACC	24	491	3.54	2	28	30
**Main effects of phase (MENS vs. POV)**							
MENS>POV	NS						
POV>MENS	pons	-	352	4.49	8	-36	-36

**Interaction (group*phase)**							
$PDM_{\Delta(MENS\text{-}POV)} > CON_{\Delta(MENS\text{-}POV)}$	NS						
$CON_{\Delta(MENS\text{-}POV)} > PDM_{\Delta(MENS\text{-}POV)}$	NS						
**Between-group planned contrast**							
MENS: PDM>CON	NS						
MENS: CON>PDM	NS						
POV: PDM>CON	NS						
POV: CON>PDM	IFG	-	335	5.06	46	20	6
	pACC	32	691	3.99	4	40	8
**Between-phase planned contrast**							
PDM: MENS>POV	NS						
PDM: POV>MENS	pons	-	325	4.58	12	-38	-34
CON: MENS>POV	NS						
CON: POV>MENS	NS						

Peak coordinates refer to Montreal Neurological Institute space. Significance was set at the uncorrected voxel level p = 0.005 followed by the FWE-corrected cluster level p = 0.05. SMA, supplementary motor area; MCC, midcingulate cortex; SFG, superior frontal gyrus; dmPFC, dorsomedial prefrontal cortex; DLPFC, dorsolateral prefrontal cortex; VPL, ventral parietal lobe; pACC, pregenual anterior cingulate cortex; IFG, inferior frontal gyrus; NS, non-significant. #: the second highest peak, almost the same location with the highest peak in the MENS phase.

Supplementary Table S4. The main effects of group and phase, interaction effect, between-group differences, and within-group differences in ReHo-seed (aIPS/SPL) functional connectivity analyses

contrast	region	BA	size	*t* score	coordinate		
					x	y	z
**ReHo-seed (aIPS/SPL) functional connectivity**							
**Main effects of group (PDM vs. CON)**							
PDM>CON	MTG	-	501	4.33	-34	-48	16
CON>PDM	NS						
**Main effects of phase (MENS vs. POV)**							
MENS>POV	NS						
POV>MENS	NS						
**Interaction (group*phase)**							
$PDM_{\Delta(MENS\text{-}POV)}$ > $CON_{\Delta(MENS\text{-}POV)}$	NS						
$CON_{\Delta(MENS\text{-}POV)}$ > $PDM_{\Delta(MENS\text{-}POV)}$	NS						
**Between-group planned contrast**							
MENS: PDM>CON	MTG	39	323	3.71	40	-60	20
MENS: CON>PDM	cerebellum anterior lobe	31	378	4.34	6	-48	-34
POV: PDM>CON	STG/MTG	22	996	4.54	56	-56	6
	MOG	19	469	4.27	-34	-80	-2
POV: CON>PDM	NS						
**Between-phase planned contrast**							
PDM: MENS>POV	NS						
PDM: POV>MENS	claustrum	-	351	4.01	-36	-8	0
	supramarginal	40	498	4.35	54	-26	18
	insula	13	434	4.16	42	2	6
CON: MENS>POV	NS						
CON: POV>MENS	NS						

Peak coordinates refer to Montreal Neurological Institute space. Significance was set at the uncorrected voxel level p = 0.005 followed by the FWE-corrected cluster level p = 0.05. MTG, middle temporal gyrus; STG, superior temporal gyrus; MOG, middle occipital gyrus; NS, non-significant.

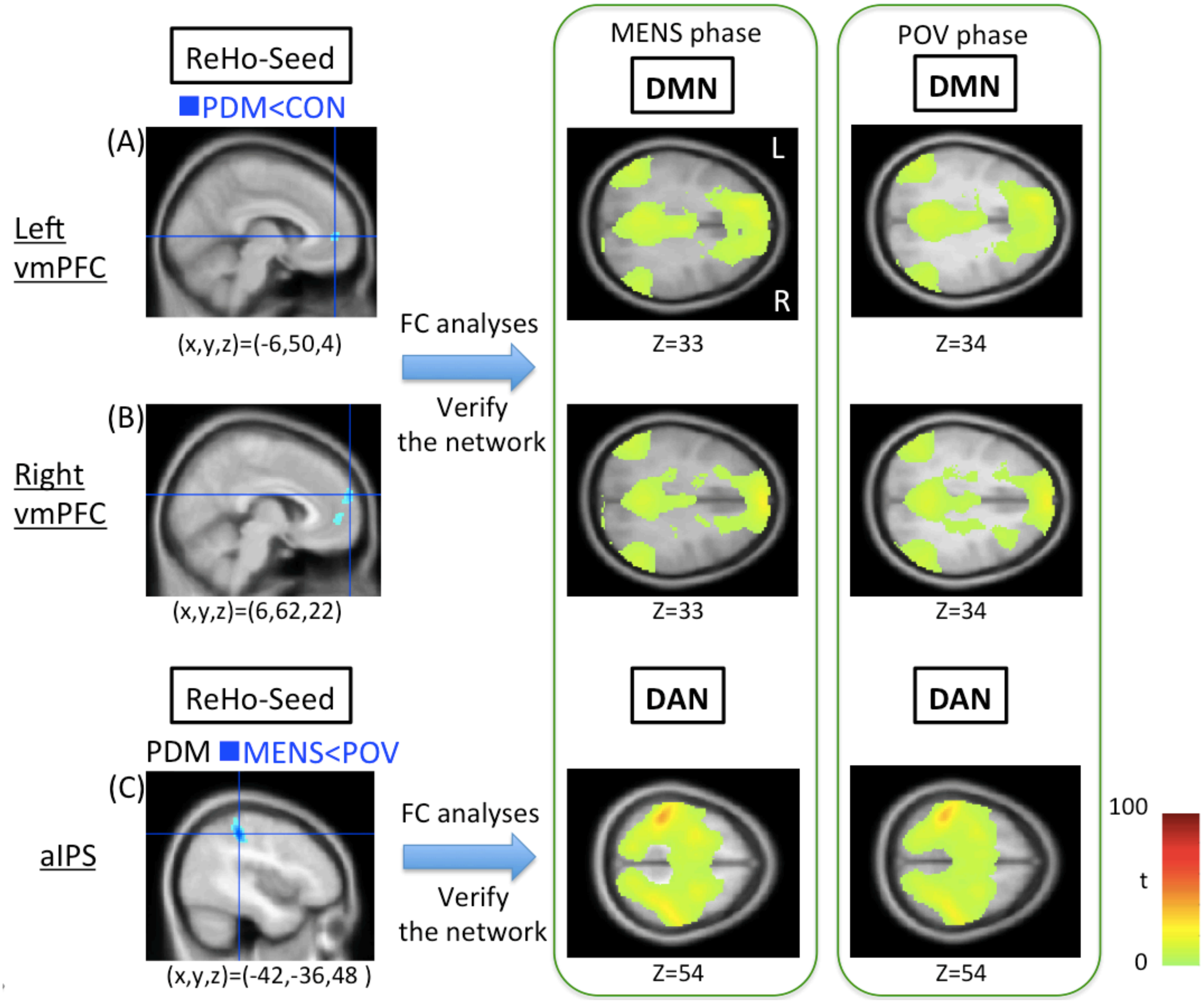

Supplementary Figure S1. Inverse validation of the networks of altered-ReHo regions

(A) and (B) The bilateral ventromedial prefrontal cortices (vmPFC) with trait-related decreased ReHo in the PDM group are neural substrates of the default mode network (DMN). (C) The left anterior part of the intraparietal sulcus (aIPS) with state-related decreased ReHo in the PDM group is a neural substrate of the dorsal attention network (DAN). The left column denotes the locations (blue crosses) of the ReHo-seeds while the middle and right columns denote the FC maps in the PDM group during MENS and POV phases, respectively. Significance was set at the FWE-corrected voxel level $p < 0.05$.

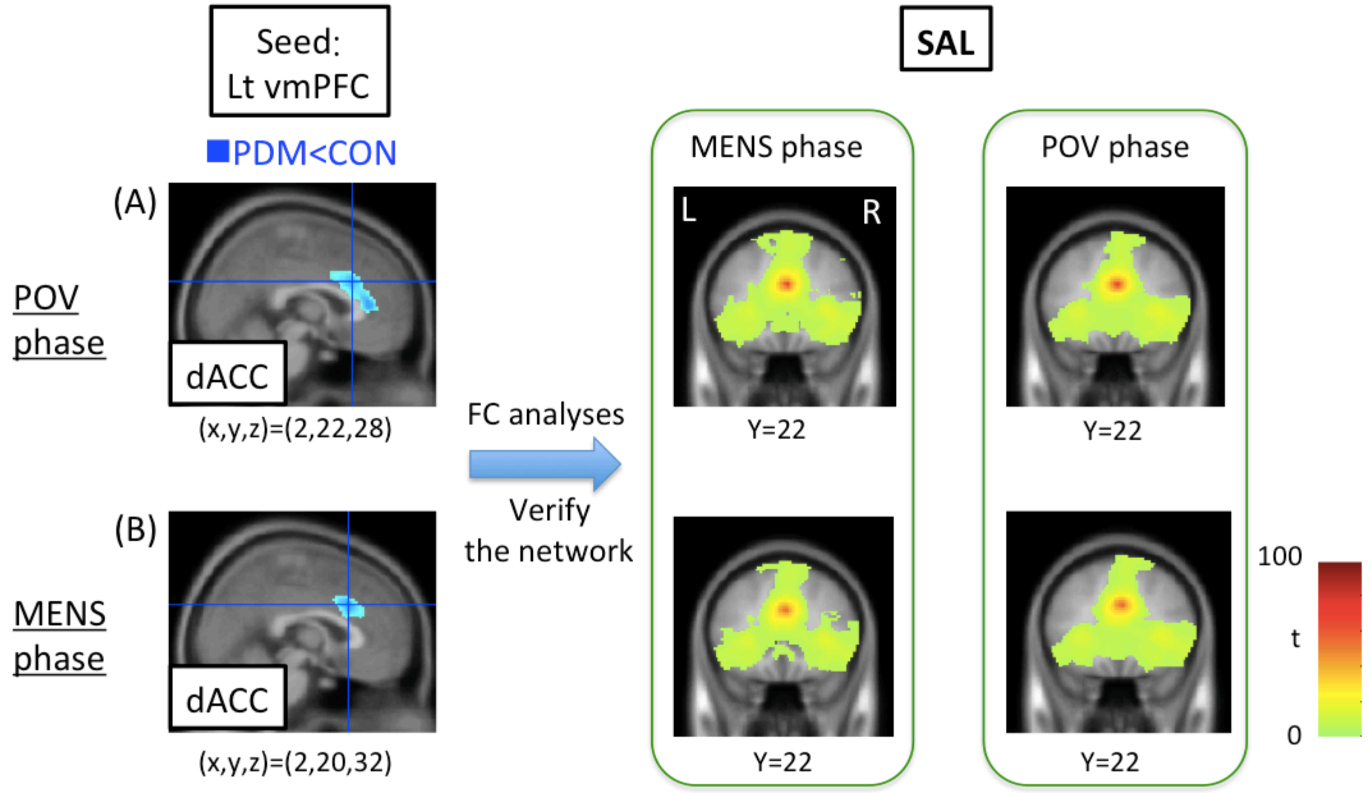

Supplementary Figure S2. Inverse validation of the networks of the regions exhibiting decreased FCs with the left ventromedial prefrontal cortex in PDM.

During the (A) periovulatory (POV) and (B) menstruation (MENS) phases, the region (dACC) exhibiting decreased FCs with the left ventromedial prefrontal cortex (Lt vmPFC) is a neural substrate of the salience network (SAL). The left column denotes the seed locations (blue crosses) of the regions exhibiting decreased FCs with Lt vmPFC while the middle and right columns denote the FC maps in the PDM group during MENS and POV phases, respectively. Significance was set at the FWE-corrected voxel level $p < 0.05$. dACC, dorsal anterior cingulate cortex

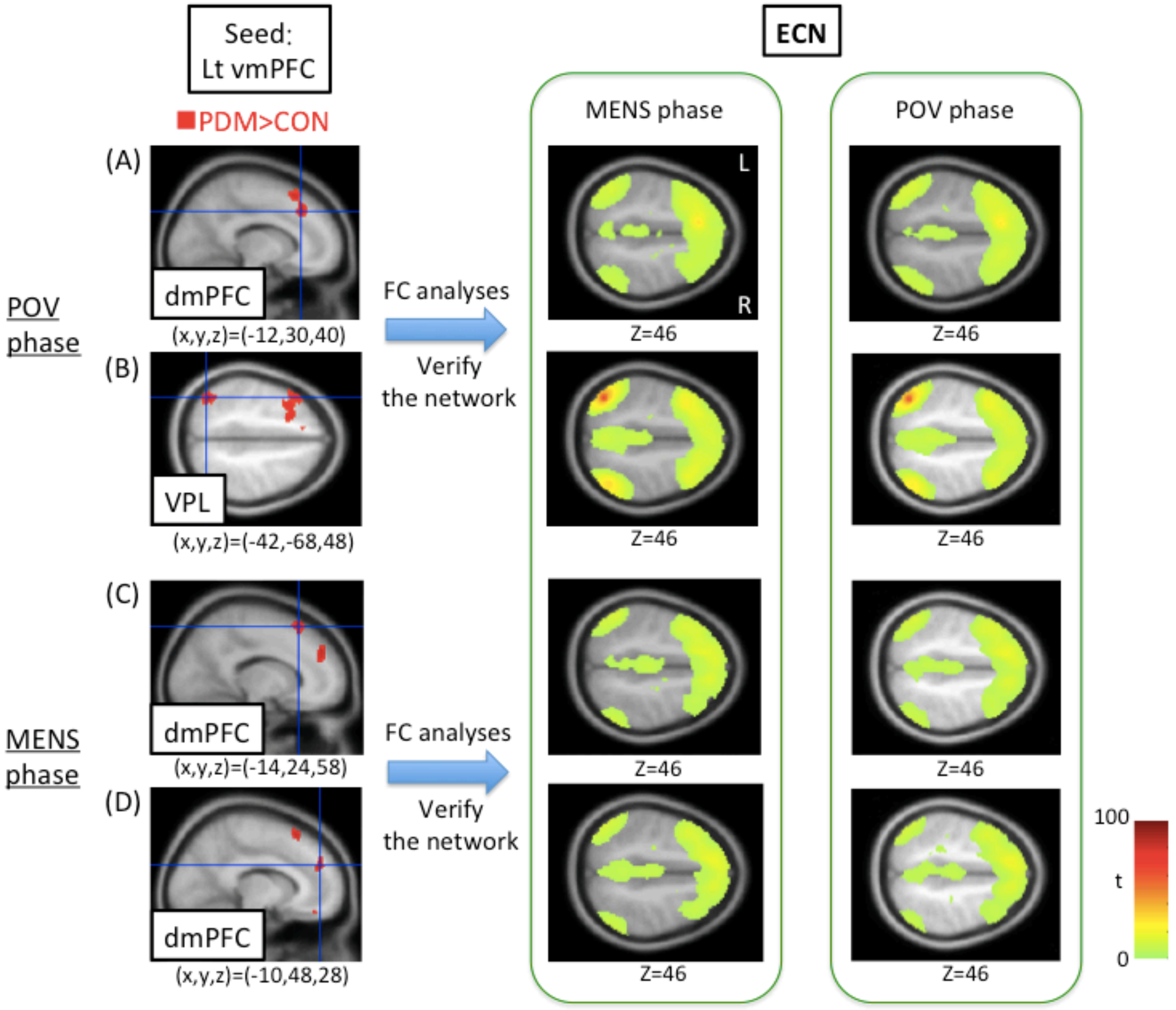

Supplementary Figure S3. Inverse validation of the networks of the regions exhibited increased FCs with the left ventromedial prefrontal cortex in PDM.

During the (A)(B) periovulatory and (C)(D) menstruation phases, the regions (dmPFC, VPL) exhibiting increased FCs with the left ventromedial prefrontal cortex (Lt vmPFC) are neural substrates of the executive control network (ECN). The left column denotes the seed locations (blue crosses) of the regions exhibited increased FCs with Lt vmPFC while the middle and right columns denote the FC maps in the PDM group during MENS and POV phases, respectively. Significance was set at the FWE-corrected voxel level $p < 0.05$. dmPFC, dorsomedial prefrontal cortex; VPL, ventral parietal lobe

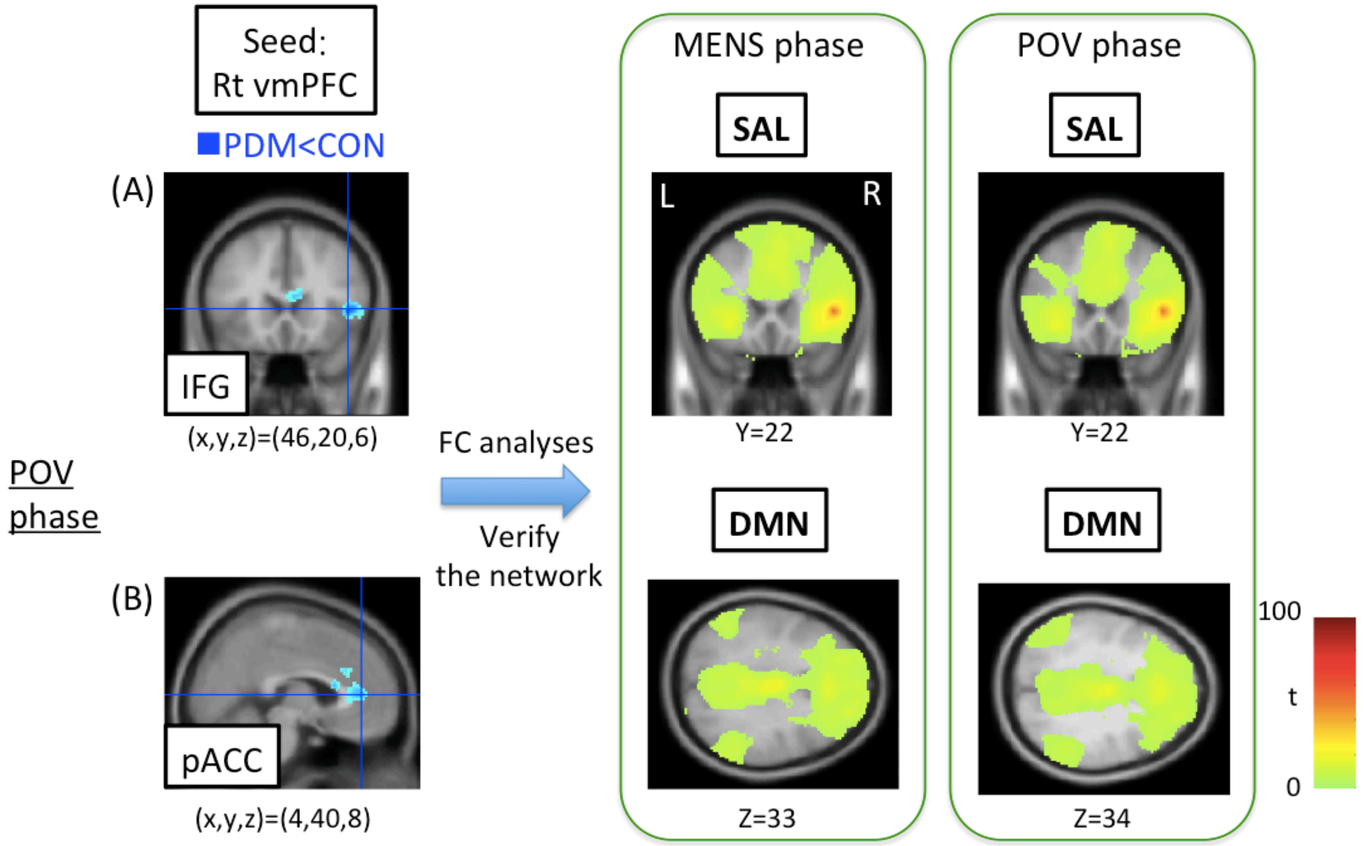

Supplementary Figure S4. Inverse validation of the networks of the regions exhibiting decreased FCs with the right ventromedial prefrontal cortex in PDM.

During the periovulatory phases, the regions (IFG, pACC) exhibiting decreased FCs with right ventromedial prefrontal cortex (Rt vmPFC) are neural substrates of the (A) salience network (SAL) (B) default mode network (DMN), respectively. The left column denotes the seed locations (blue crosses) of the regions exhibiting decreased FCs with Rt vmPFC while the middle and right columns denote the FC maps in the PDM group during MENS and POV phases, respectively. Significance was set at the FWE-corrected voxel level $p < 0.05$. IFG, inferior frontal gyrus; pACC, pregenual anterior cingulate
